# Sound environment in an urban apartment building during and after the COVID-19 lockdown

**DOI:** 10.3389/fpsyg.2023.1136201

**Published:** 2023-03-02

**Authors:** Tingting Yang, Jian Kang

**Affiliations:** ^1^Department of Architecture, College of Architecture and Urban Planning, Tongji University, Shanghai, China; ^2^Institute for Environmental Design and Engineering, The Bartlett, University College London, London, United Kingdom

**Keywords:** COVID-19 lockdown, urban community, sound environment, noise pollution, quiet community

## Abstract

Quiet areas, such as quiet communities, are encouraged to maintain a harmonious and peaceful urban living environment, and the design approach has drawn increasing attention in recent years. Related residential standards define the thresholds of quietness concerning noise pollution problems. However, the variations in height across floors of high-rise buildings and time in sound environments have not been detailed. The city of Shanghai experienced a citywide lockdown to contain the spread of COVID-19 showing the evidence of quietness with marked reductions in anthropogenic noise. Here, we conducted noise monitoring in a 14-story apartment building surrounded and shielded by other buildings in a typical urban community during and after lockdown. The mean value of the equivalent continuous A-weighted sound pressure level (*L*_*Aeq*_) of all 14 floors after lockdown was higher than that during lockdown, and the differences were 3.6 and 3.1 dB during the daytime and night-time periods, respectively. The *L*_*Aeq*_ values at low heights were slightly lower than those at high heights during and after lockdown. The variations due to the different heights were not great. However, the change tendency from the ground to the top floors was similar and correlated during and after lockdown. The difference between the maximum and minimum values of the floors was 3 dB during the daytime period and 4.5 to 5.1 dB during the night-time period. The day equivalent continuous A-weighted sound pressure level (*L*_*day*_) and night equivalent continuous A-weighted sound pressure level (*L*_*night*_) at the middle building height increased 4.0 and 1.3 dB, respectively, after lockdown. The *L*_*Aeq*_ change tendency during a daily cycle during and after lockdown was similar and highly correlated. The differences in the frequency characteristics of noise level were larger within the 63 to 2,000 Hz range. We suggest that the building represents a typical quiet living condition in high-density habitats in China. Notably, the difference is approximately 3 to 4 dB, and the patterns of variation in height and time are similar between the absence and limited presence of anthropogenic noise. In practice, it would be useful to consider specific floor level or time of day.

## 1. Introduction

Noise pollution is a widely recognized problem for most worldwide high-density cities (World Health Organization [WHO], 2018). Control of this issue has become increasingly necessary. Mounting evidence over the past decades indicate a strong association between the noise and wellbeing concerning psychological and health issues. Sounds exceeding 50 dBA may cause annoyance, disturbed sleep, delirium and elevations in blood pressure and are linked to ischemic heart disease in healthy populations (Morrison et al., 2003). Actions implemented to maintain a harmonious and peaceful living environment include the promulgation of the noise pollution prevention and control law of the People’s Republic of China in National People’s Congress Standing Committee (2022), which encourages the creation of quiet areas, e.g., quiet communities and cabins. Increasing attention has been given to understanding the impacts of quiet communities on wellbeing.

Due to the threats of the coronavirus disease 2019 (COVID-19) pandemic to human life and welfare, Shanghai, a typical high-density city in China, with a total area of 6340.5 km^2^ and a resident population of around 25 million at the end of 2020, initiated a citywide lockdown from 1 April to 31 May of 2022. The suspension of all transportation networks and use of public spaces, as well as stay-at-home orders, have inadvertently resulted in a natural experiment, not only presenting vital insights and objective evidence of quiet areas, but also enabling the comparison between quiet and noisy sound environments in the same area. There has been research showing evidence by noise measurements in urban environments in many cities during the lockdown, for example, an average reduction of 5.4 dB [equivalent continuous A-weighted sound pressure level (*L*_*Aeq*_)] in 11 locations in London (Aletta et al., 2020), a sound level reduction ranging from 4 to 5 dBA in Madrid (Asensio et al., 2020) and a considerable sound energy decrease in 5 locations in Buenos Aires (Said et al., 2020). Evidence regarding quiet urban communities remains to be specified.

In related residential standards in regard to the noise pollution problem in urban communities, thresholds for daytime and night- time noise levels in both outdoor and indoor environments have been separately defined (Ministry of Environmental Protection, 2008; Ministry of Justice of the People’s Republic of China, 2021). In these standards, variations in height and time in sound environments have not been detailed. Previous studies have presented evidence on the effect of traffic noise on the variations due to height and time by measurements and simulations in urban communities (Xia et al., 2007; Lou and Ma, 2014). For example, the noise level increased with height from the 1st floor to the 6th floor and then decreased (Li et al., 2012). These studies demonstrated that the sound environment in urban communities is more complicated. To better understand the effects of the reduction in anthropogenic noise from a psychological view, it would be essential to obtain a more comprehensive overview of relevant features in the physical environment.

The aim of this study was to explore (1) whether there are variations in height in the sound environment either during or after lockdown; (2) whether there are variations in time in the sound environment either during or after lockdown; and (3) whether there are differences in noise levels between during and after lockdown in urban communities. Noise monitoring measurements were conducted in a typical urban community during and after lockdown for comparisons, and suggestions for the design of quiet urban communities in high-density habitats were formulated.

## 2. Materials and methods

### 2.1. Site selection

**FIGURE 1:**
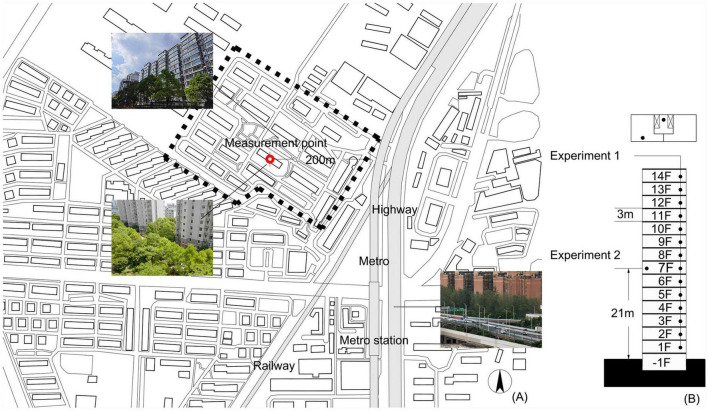
Details of the site and environmental conditions. **(A)** Plan showing the location of the building for measurements (red point); **(B)** section showing the experimental measurement points.

The building used for the measurements was in the center of an urban community surrounded and effectively shielded by other buildings, which were located in the northern area of Puxi district in Shanghai constructed in 2012 as shown in Figure 1. The residential area covered 76,790 m^2^ with a plot ratio of 2.0, which is not uncommon in the cities of China. Normally, outside the area of the community, there were four transportation systems including a railway, highway, metro and roads, constantly contributing to traffic noise affecting the sound environment of the site. None of the transportation systems was operational during the lockdown, and they returned to normal at the same time on 1 June of 2022, i.e., the official announcement of the end of lockdown.

The building was chosen as a typical site in high density cities, in terms of its dimensions, height, location, and the building density of the area measured by the plot ratio. The shielding of surroundings, including other buildings and green spaces, attenuates traffic noise from the metro and highway, which could avoid effects caused by certain specific environments. For more specific cases, e.g., the plot ratio exceeds the recommended value, or the community is located in a large city park, further studies could be made, also by using computer simulations.

All apartment buildings in the community, including the one for measurements, were 14-story slab-type buildings with an equivalent floor height of 3 m, as shown in the section of Figure 1B. The windows of the individual rooms of each apartment faced either northward or southward. In addition, the measured building was surrounded by the green spaces. The greening ratio of the community was documented as 30%. The vegetation provided a suitable bird habitat, which mainly comprises camphor trees, shrubs and grass. The vegetation height ranged from approximately 6 to 18 m, which was close to the height of the 3rd and 6th floors.

Along the vertical direction, the apartment building was divided into four sections in accordance with the related floor-specific standard of urban communities, namely, the 1st to 3rd floors (from 0 to 9 m) were categorized as the low section, the 4 to 6th floors (from 9 to 18 m) were categorized as the middle section, the 7 to 9th floors (from 18 to 27 m) were categorized as the middle/high section, and the 10th to top floors (from 27 to 42 m) were categorized as the high section.

### 2.2. Experimental design

We conducted both short-term and long-term noise monitoring during and after lockdown. For the convenience of comparisons between and across the results, the measurement parameter was *L*_*Aeq*_ and all the time lengths of each noise level measurement lasted for 1 min, which was accomplished in the outdoor environment with an open window. For the classification of times of a daily cycle, we divided the time at 1-h intervals and further divided the 24 h into two sections: daytime (06:00–22:00) and night-time (22:00–06:00) periods. All the measurements in this research were conducted in one apartment building.

Experiment 1 (effects of height): The site comprised 14 floors, and therefore, there were 14 measurement points located at the same position on each floor, namely, in the public space outside the apartments open to the elevators and staircases as shown in Figure 1B. The window of each public space faced northward. Due to the restrictions of the lockdown policy, the measurements on each floor were conducted by one researcher in descending order from the 14th to 1st floors. The measurement time during the daytime was divided into three sections, representing the morning (08:00–10:00), noon (12:00–14:00), and evening (18:00–20:00) periods. The measurement time at night ranged from 23:00 to 01:00. Consequently, we conducted four measurements during these four time periods on each floor throughout the daily cycle. The measurements during lockdown were conducted in the first week of April 2022, and those after lockdown were conducted in the first week of December 2022.

Experiment 2 (effects of time): Long-term noise monitoring lasted 24 h to cover both daytime and night-time periods. The measurement point was located on the 7th floor, i.e., at the middle height of the apartment building, on the balcony of a living room. The measurements during lockdown were conducted in the first week of April 2022, and those after lockdown were conducted in the first week of June 2022.

We used an AWA6228 + class 1 sound level meter (Aihua, Hangzhou, China) to measure the noise level, which was associated with a type-1 microphone with a frequency range of 10 to 20 kHz. All recordings were conducted at the measurement points described above according to standard procedures (Ministry of Environmental Protection, 2008; Ministry of Justice of the People’s Republic of China, 2021).

## 3. Results

### 3.1. General effects

To explore the difference between during and after lockdown, we conducted noise measurements on 14 separated floors at different time periods during a daily cycle. Table 1 shows the mean values and standard deviations of *L*_*Aeq*_ of different height sections, i.e., low, middle, middle/high and high sections.

**TABLE 1:** Comparison of the mean value and standard deviation of *L*_Aeq_ between the various height sections during and after lockdown.

Height of the sections	During lockdown		After lockdown		Difference	
	Day	Night	Day	Night	Day	Night
High	47.4 ± 0.6	43.4 ± 0.6	50.6 ± 0.7	46.6 ± 0.5	3.2	3.2
Middle/high	46.7 ± 0.4	42.1 ± 0.2	49.9 ± 0.5	46.1 ± 0.4	3.2	4.0
Middle	45.7 ± 0.8	42.1 ± 0.5	49.8 ± 0.7	44.9 ± 1.3	4.1	2.8
Low	45.8 ± 0.7	40.9 ± 1.0	49.6 ± 1.0	43.4 ± 0.8	3.8	2.5
Mean	46.5 ± 1.0	42.3 ± 1.1	50.1 ± 0.8	45.4 ± 1.3	3.6	3.1

The mean value of *L*_*Aeq*_ of all 14 floors after lockdown was much higher than that during lockdown. For the daytime period, during lockdown, the mean value of *L*_*Aeq*_ of all 14 floors was 46.5 dBA, and the standard deviation was 1.0 dB during lockdown. Comparatively, after lockdown in December, the mean value of *L*_*Aeq*_ was 50.1 dBA, and the standard deviation was 0.8 dB. As a result, the difference between the mean value of *L*_*Aeq*_ during and after lockdown was 3.6 dB. For the night-time period, during lockdown, for all 14 floors, the mean value of *L*_*Aeq*_ was 42.3 dBA, and the standard deviation was 1.1 dB. Comparatively, after lockdown in December, the mean value of *L*_*Aeq*_ was 45.4 dBA, and the standard deviation was 1.3 dB. As a result, the difference between the mean value during and after lockdown was 3.1 dB. In addition, according to the results of the *t*-test between the groups during and after lockdown in the daytime and night-time periods, statistically significant differences (*p* = 0.000) existed between these two groups.

### 3.2. Effects of height

**FIGURE 2:**
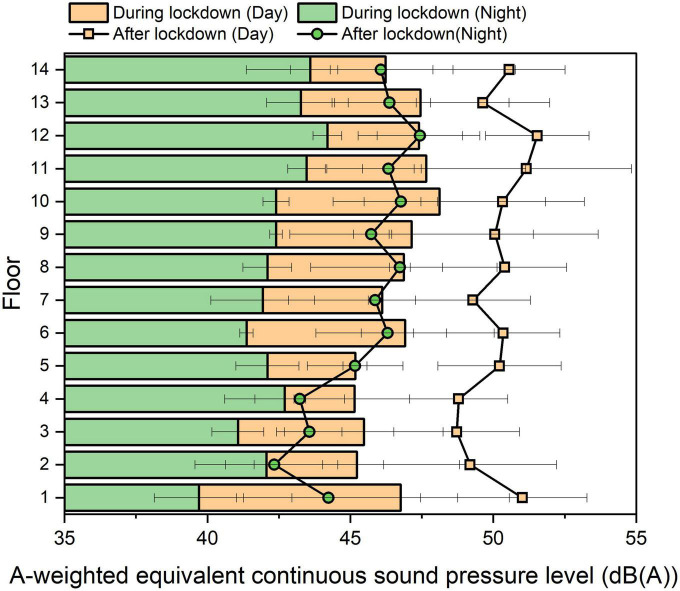
Comparison of the mean value and standard deviation of LAeq measured on each of 14 floors during and after lockdown. The daily cycle of 24 h was divided into daytime (06:00–22:00) and night-time (22:00–06:00) periods.

Figure 2 shows the mean value and standard deviation of *L*_*Aeq*_ obtained of all 14 floors, as measured in the public space of every floor with an open window. The bar denotes the results during lockdown in April, and the line denotes the results half a year after lockdown ended in December.

We found that for both during and after lockdown, the mean values at low heights were slightly lower than those at high heights, as summarized in Table 1. This indicated an even quieter environment during and after lockdown on the low height section rather than high height section, probably because of the sound absorption by the surrounding vegetation on the ground. This corresponds to the results obtained in the previous research which also shows the absorption effects on the ground (Li et al., 2012). In addition, higher floors also have less obstructed sound paths to noise sources that are not in close proximity of the building.

For the daytime period, according to the results of the ANOVA tests between the groups of 14 separated floors, no significant difference was found either during or after lockdown (*p* = 0.857 and *p* = 0.709), indicating that the difference between 14 separated floors either during or after lockdown was not notable. However, according to the results of ANOVA tests between the groups of different time periods in the daytime, i.e., in the morning (08:00–10:00), noon (12:00–14:00), and evening (18:00–20:00), there were statistically significant differences either during or after lockdown (*p* = 0.000, and *p* = 0.000), demonstrating that the difference in the mean *L*_*Aeq*_ value of 14 separated floors between different time periods during a daily cycle was notable. Therefore, it is concluded that the variations due to the height in the sound environment were small, while those due to the time were large during the daytime period in this apartment building. This probably occurs because during lockdown, the sound environments were not affected by the traffic noise but were dominated by natural sound.

**FIGURE 3:**
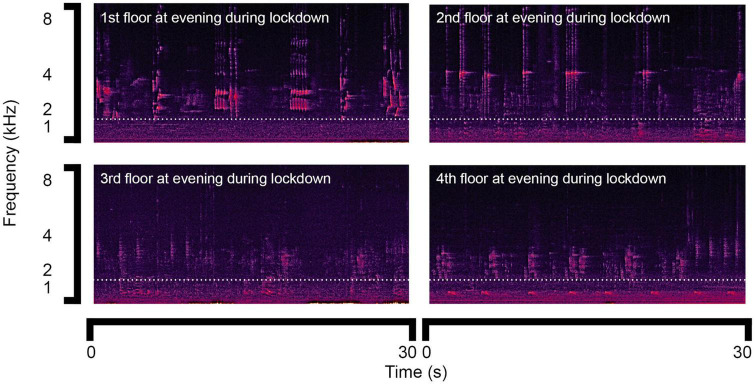
Sound spectrograms of bird song during lockdown.

For the observation of the natural sound, Figure 3 shows the sound spectrograms of bird song lasting for 30 s in the evening around 18:00 during lockdown obtained on the 1st, 2nd, 3rd, and 4th floors, which were collected continuously from the 4th to 1st floors within 15 min. The diversity of bird song in the community was demonstrated by the variations during a short period. The bird song frequency ranged from 1 to 8 kHz.

**FIGURE 4:**
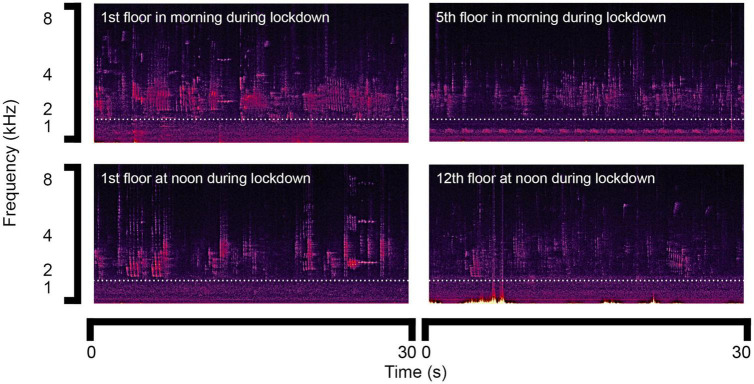
Sound spectrograms of bird song in the morning and at noon during lockdown.

Furthermore, we compared the results of bird song lasting for 30 s in the morning around 08:00 during lockdown on the 1st and 5th floors (Figure 4). Differences in frequency characteristics were not observed. We further compared the sound spectrograms obtained at noon around 12:00 during lockdown on the 1st and 12th floors. The results turned out to be similar as shown in Figure 4. Therefore, it is concluded that the differences in the frequency characteristics of bird song during lockdown between the different floors were insignificant.

**FIGURE 5:**
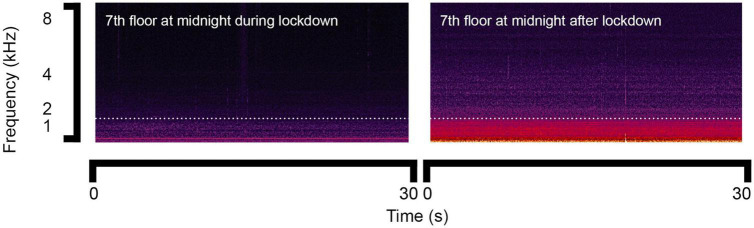
Sound spectrograms of noise at midnight on the 7th floor during and after lockdown.

Comparatively, during the night-time period, according to the results of the ANOVA test between the groups of 14 separated floors, there was no statistically significant difference either during or after lockdown (*p* = 0.190 and *p* = 0.205). Furthermore, according to the results of ANOVA tests between the groups of values measured obtained at night on the 14 separated floors, there was also no statistically significant difference during or after lockdown (*p* = 0.651 and *p* = 0.087), which indicates that the effects of both height and time were not notable during the night-time period. Figure 5 shows the comparison of sound spectrograms at midnight. A large increase could be observed in the low frequency range due to the traffic noise after lockdown.

By conducting the correlation analysis of the change tendency on all 14 floors between the periods during and after lockdown, as shown in Figure 2, we obtained correlation coefficients of 0.6 and 0.5 during the daytime and night-time periods, respectively, representing an association between the periods during and after lockdown. This demonstrated that although there were distinct level differences in between, the change tendency on all 14 floors during and after lockdown was similar, which presents a quiet environment of urban community different from those significantly affected by the traffic noise (Xia et al., 2007; Lou and Ma, 2014).

Considering the effects of traffic noise during and after lockdown, the maximum difference during and after lockdown in the daytime period was obtained on the 5th floor at 5.0 dB, and the minimum difference was obtained on the 10 and 13th floors at approximately 2.2 dB. In addition, we found that the maximum difference during and after lockdown in the night-time period was obtained on the 6th floor at 4.9 dB, and the minimum difference was obtained on the 2nd and 4th floors at approximately 0.4 dB. In this case, although traffic considerably increased *in situ*, the same increase did not occur on every floor.

We found that during lockdown, the maximum *L*_*Aeq*_ value of 48.1 dBA was obtained on the 10th floor, and the minimum value of approximately 45 dBA was measured across the 2nd to 5th floors during the daytime period, reflecting a level difference of 3 dB among the 14 separated floors; during the night-time period, the maximum and minimum values were obtained on the 12th floor for 44.2 dBA and the 1st floor for approximately 39.7 dBA, respectively, showing a level difference of 4.5 dB of the apartment building.

Comparatively, after lockdown, the maximum value of of 51.5 dBA was obtained on the 12th floor, and the minimum value of approximatively 49 dBA was found to be across the 2nd to 4th floors during the daytime period; therefore, there was a level difference within 3 dB. During the night-time period, the maximum and minimum values were obtained on the 12th floor for approximately 47.4 dBA and the 2nd floor for approximately 42.3 dBA, presenting a level difference of 5.1 dB among the 14 separated floors. These variations may be due to the effects of wind noise on the vertical distribution of the apartment building.

It is interesting to note that during lockdown, the mean value of the level difference between the daytime and night-time periods on every floor was 4.2 dB, and the standard deviation was 1.3 dB. The largest difference of 7.1 dB was observed on the 1st floor and the smallest difference of approximately 2.5 dB was observed on the 4th floor and 14th floors. Comparatively, after lockdown, the mean value of the level difference between the daytime and night-time periods on every floor was 4.7 dB, and the standard deviation was 1.1 dB. The largest differences of approximately 6.8 dB were also found on the 1st and 2nd floors and the smallest differences were also found on the 13th floor. The results both during and after lockdown exhibited statistically significant differences (*p* = 0.000) according to the results of *t*-tests between the groups of *L*_*Aeq*_. Therefore, we suggest that the difference in the sound environment between the daytime and night-time periods was much larger at low heights than at high heights.

### 3.3. Effects of time

To further explore the effects of time, the data presented here is for the 7th floor, and the 24-h period could be further analyzed by considering the daytime and night-time periods separately.

First, during the daytime period, the day equivalent continuous A-weighted sound pressure level (*L*_*day*_) reached 43.7 and 47.7 dBA during and after lockdown, respectively. Therefore, the *L*_*day*_ level difference between during and after lockdown reached 4.0 dB. As expected, the effects of traffic flow on this apartment building were considerably reduced during lockdown. However, it is not possible to establish a precise quantitative relationship between the absence of anthropogenic noise and vehicle flow reduction during and after lockdown due to the complexity of traffic systems outside the community.

Comparatively, during the night-time period, the night equivalent continuous A-weighted sound pressure level (*L*_*night*_) was 42.8 and 44.1 dBA during and after lockdown, respectively. Therefore, the level difference between the periods during and after lockdown in the community was only approximately 1.3 dB, which was much smaller than that of *L*_*day*_, indicating a comparable and quiet environment in this community at night after lockdown.

**FIGURE 6:**
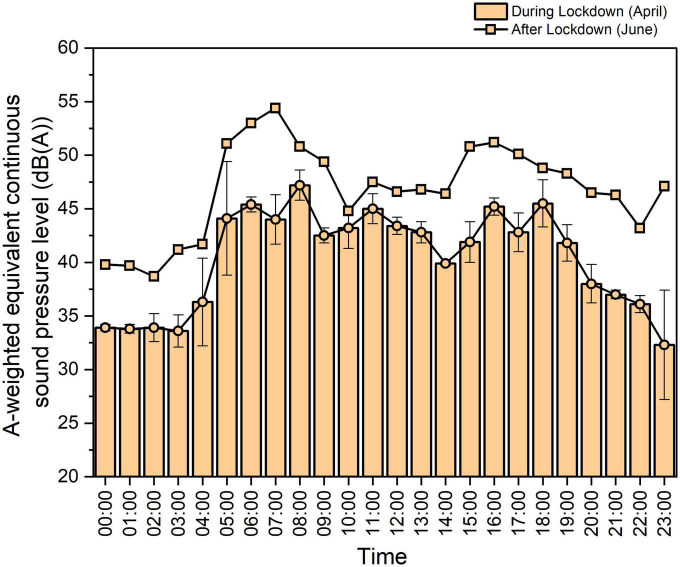
Comparison of the mean value of *L*_Aeq_ over 24 h measured during lockdown in April and after lockdown in June showing the effect of time during a daily cycle.

**FIGURE 7:**
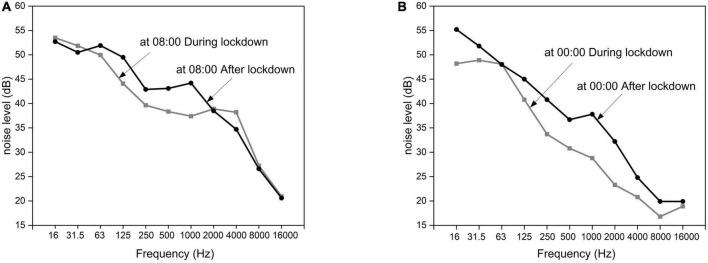
Difference in the frequency characteristics during and after lockdown. **(A)** At 08:00; **(B)** at 00:00.

Figure 6 shows the mean value and standard deviation of *L*_Aeq_ measured on the seventh floor during lockdown in April and after lockdown in June. We found that during lockdown, the *L*_Aeq_ values generally ranged from 35 to 45 dB from 06:00 to 22:00 in the daytime. The highest values were observed at 08:00, and the lowest values were observed at 14:00, as shown by the bars. The standard deviations of the noise level were smaller than 2 dB at most times of the day and night, except for 04:00, 05:00, 07:00, 18:00, and 23:00. The greatest level increase after lockdown of approximately 12.0 dB was found between 05:00 and 07:00 and the smallest increase of approximately 0.3 dB occurred from approximately 10:00 to 11:00 during the daytime. A very large difference was observed at 23:00. According to the observation of the researcher, this is probably due to the traffic noise generated by the passing train on the nearby railway, which occurred regularly at this particular time every day after lockdown. Figure 7 shows the difference in the frequency characteristics of noise level during and after lockdown at 08:00 and 00:00. The differences in the frequency characteristics of noise level were notable within the 63 to 2,000 Hz range.

The temporal changes in *L*_Aeq_ values during a daily cycle during lockdown reflected the considerable reduction in anthropogenic noise and were more notably related to the rhythm of natural sources. We conducted a correlation analysis of the change tendency during the daily cycle from April to June and obtained a correlation efficient of 0.8, suggesting a high association between during and after lockdown. This demonstrated that although there were distinct level differences between these periods due to the increase in anthropogenic noise, this did not impact the tendency during a daily cycle.

## 4. Discussion

As discussed in (Section “2.1. Site selection”), the building used for measurements was a typical living condition in high-density habitats in China. The reduction in noise level during and after lockdown in London, e.g., an average 5.4 dBA (Aletta et al., 2020), was larger than the reduction in this study. This is owing to the influence by the specific environments considered. To consider other conditions, further work should be carried out, also with the help of computer simulation of sound propagation.

While the work reported in this paper is mainly on the objective fact, it is also useful to interpret the results from a psychological point of view, e.g., studies in London (Torresin et al., 2021, 2022). First, we found that the differences in noise level were 3 to 4 dB during and after lockdown for both daytime and night-time periods. Because 3 dB is the threshold of perception, the difference could be barely perceptible.

Secondly, the variations due to the height were demonstrated to be not great, which indicates that the difference in perception, such as annoyance, of the residents across floors of the same high-rise building could be small. Furthermore, the *L*_Aeq_ values at low heights were slightly lower than those at high heights. The changing tendencies from the bottom to top floor were similar and correlated during and after lockdown, indicating that the pattern was not affected by limited increase in anthropogenic noise. Therefore, the perception of the residents at different floors of the building could be divided into groups, exhibiting different features. Designers could analyze and design the sound environment in a partitional way when necessary.

Thirdly, because the change tendency of *L*_Aeq_ during a daily cycle was similar during and after lockdown, indicating a stable pattern in the case of limited increase in anthropogenic noise, at least from the start of April to the end of May. Therefore, people can take advantage of the sound environment at certain time of the day for regular day-based activities, such as restorative rehabilitation, in quiet communities.

## 5. Conclusion

Under the condition of the absence or presence of anthropogenic noise, we explored the variations in the sound environment during and after lockdown by conducting noise monitoring measurements concerning the differences due to height and time in a 14-floor apartment building in a common urban community. It is concluded that

1. The mean value of *L*_Aeq_ all 14 floors after lockdown was higher than that during lockdown, and the differences were 3.6 and 3.1 dB during the daytime and night-time periods, respectively.
2. For both during and after lockdown, the values at low heights were slightly lower than those at high heights. The variations due to the height were not great, while the mean value of all 14 floors among different times in the daytime fluctuated; however, the change tendency from the ground to top floor was similar and correlated during and after lockdown. The difference between the maximum and minimum values across all floors was 3 dB during the daytime period and 4.5 to 5.1 dB during the night-time period.
3. The difference between the daytime and night-time periods was evident, which was largest on the ground floor and smallest on the top floor. Compared to the results during lockdown, the *L*_day_ and *L*_night_ values at a location at middle height of the apartment building increased 4.0 and 1.3 dB, respectively, after lockdown. The same increase did not occur every time; however, the change tendency during a daily cycle during and after lockdown was similar and highly correlated. The differences in the frequency characteristics of noise level were larger within the 63 to 2,000 Hz range.

The building in this research represents a typical quiet living condition in high-density habitats in China. Notably, the difference is approximately 3 to 4 dB, and the patterns of variation in height and time are similar between the absence and presence of anthropogenic noise. In practice, it would be useful to consider specific floor level or time of day.

## Data availability statement

The original contributions presented in this study are included in this article/supplementary material, further inquiries can be directed to the corresponding author.

## Author contributions

TY and JK: conceptualization and writing – reviewing and editing. TY: writing – original draft. JK: supervision. Both authors contributed to the article and approved the submitted version.
